# Evaluation of the Therapeutic Potential of Amantadine in a Vincristine-Induced Peripheral Neuropathy Model in Rats

**DOI:** 10.3390/ani14131941

**Published:** 2024-06-30

**Authors:** Isabela Santana Albertazzi Drummond, Jéssica Natália Silva de Oliveira, Raquel Vieira Niella, Álvaro José Chávez Silva, Iago Santos de Oliveira, Sophia Saraiva de Souza, Claire Souza da Costa Marques, Janaina Maria Xavier Corrêa, Juneo Freitas Silva, Mário Sérgio Lima de Lavor

**Affiliations:** 1 Department of Agricultural and Environmental Sciences, State University of Santa Cruz (UESC), Ilhéus 45662-900, BA, Brazil; 2 Department of Biological Sciences, State University of Santa Cruz (UESC), Ilhéus 45662-900, BA, Brazil

**Keywords:** amantadine, cancer, neuroinflammation, nociception, NMDA, oxidative stress

## Abstract

**Simple Summary:**

Vincristine-induced peripheral neuropathy is a debilitating side effect and a limiting factor in the continuance of treatment of cancer patients. Thus, therapeutic strategies are necessary to enable the maintenance of oncological treatment and minimize undesired conditions caused by the treatment. Current protocols for the management of neuropathic pain remain unsatisfactory due to the complexity and severity of clinical signs, as well as the lack of significant improvement with pharmacological treatment. Amantadine shows therapeutic potential by acting on NMDA receptors. Immunohistochemistry, quantitative PCR and analysis of enzymatic activity were performed to demonstrate the therapeutic activity of amantadine in the spinal cord of rats with induced neuropathic pain. The authors herein demonstrated positive effects on the regulation of neuroinflammation, oxidative stress, reticulum endoplasmic stress and apoptosis with the highest doses examined (25 mg/kg and 50 mg/kg). We hope that our research will contribute to studies of amantadine as an option in a pharmacotherapeutic protocol for peripheral neuropathy.

**Abstract:**

This study aimed to evaluate the therapeutic potential of amantadine in a vincristine-induced peripheral neuropathy model in rats. Forty-eight male Wistar rats were used. The treated groups received oral amantadine at doses of 2, 5, 12, 25 and 50 mg/kg, with daily applications for 14 days. The mechanical paw withdrawal threshold was measured using a digital analgesimeter. Immunohistochemical analysis of IL-6, TNFα, MIP1α, IL-10, CX3CR1, CXCR4, SOD, CAT and GPx, and enzymatic activity analysis of CAT, SOD and GPx were performed, in addition to quantitative PCR of *Grp78*, *Chop*, *Ho1*, *Perk*, *Bax*, *Bcl-xL*, *Casp 3*, *Casp 9*, *IL-6*, *IL-10*, *IL-18* and *IL-1β*. The results showed an increase in nociceptive thresholds in animals that received 25 mg/kg and 50 mg/kg amantadine. Immunohistochemistry showed a decrease in the immunostaining of IL-6, TNFα, MIP1α and CX3CR1, and an increase in IL-10. CAT and SOD showed an increase in both immunochemistry and enzymatic analysis. qPCR revealed a reduced expression of genes related to endoplasmic reticulum stress and regulation in the expression of immunological and apoptotic markers. Amantadine demonstrated antinociceptive, anti-inflammatory and antioxidant effects in the vincristine-induced peripheral neuropathy model in rats, suggesting that amantadine may be considered an alternative approach for the treatment of vincristine-induced peripheral neuropathic pain.

## 1. Introduction

Cancer is one of the main causes of decreased quality of life worldwide, with a high death rate [1]. In the last global estimate carried out by Globocan in 2020, it was estimated that around 19.3 million new cases of cancer were identified in 2020 alone and approximately 10 million cancer deaths in that same year [2,3]. The World Health Organization, in 2019, estimated that, in 112 countries, cancer was the first or second leading cause of death for people under 70 years of age, and in a further 23 countries it ranked third or fourth [4]. In the treatment of cancer patients, chemotherapy is one of the methods used, in addition to radiotherapy, surgery and supportive treatments. Chemotherapy drugs routinely used in the clinic are not pharmacologically selective, which means that they interact non-specifically on tumor and healthy cells, causing adverse effects on the latter [5].

Among chemotherapy drugs of great importance in medicine, vincristine is used in several protocols for various cancers, such as acute leukemia, Hodgkin’s disease, neuroblastoma, uterine cervix carcinoma, lymphomas, breast cancer and melanoma, and is also used in therapeutic protocols for other neoplasms in association with additional chemotherapy agents [6]. The most commonly observed adverse effects induced by this drug include peripheral neuropathy, paresthesia, gastrointestinal tract disorders, namely anorexia, diarrhea and vomiting, alopecia and mild immunosuppression [7]. Most patients treated with vincristine with a cumulative dose greater than 4 mg/m^2^ develop some degree of sensory peripheral neuropathy [8].

Peripheral neuropathy is the most common neurological syndrome secondary to antineoplastic therapy, and occurs in a dose-dependent manner, which can disrupt the chemotherapy protocol by leading to drug changes, dose reductions and even discontinuation of treatment [9]. Of cancer patients undergoing treatment, more than 60% have peripheral neuropathy linked to the use of chemotherapy drugs, with chemotherapy-induced peripheral neuropathy (CIPN) being one of the most common adverse effects of chemotherapy [10]. Its pathophysiology is multifactorial and involves neuroinflammation, immunological processes, oxidative stress, apoptotic mechanisms, changes in calcium homeostasis, axon degeneration and membrane remodeling [11].

The treatment of neuropathic pain is still uncertain and is still far from satisfactory, as most of those patients do not achieve significant improvement with pharmacological treatment, and the clinical signs of neuropathic pain can be severe and respond poorly to analgesic therapeutic protocols [12]. Opioids were used as first-line agents, but they still did not reach an attractive therapeutic significance [13]. Soon after, anticonvulsants were added to the class of first-line medications, but their effectiveness was still limited. Currently, non-steroidal anti-inflammatory drugs (NSAIDs), alpha-2-adrenergic agonists, antidepressants and NMDA antagonists are most commonly used. Studies suggest interest in the therapeutic efficacy in neuropathic pain of N-methyl-D-aspartate (NMDA) receptor antagonists [14,15,16].

Amantadine, a drug initially used as an antiviral and for the treatment of the influenza virus and in the treatment of Parkinson’s, is also being considered for pain management, since its mechanism of action promotes the non-competitive antagonism of glutamatergic receptor damage of the NMDA type, which participates in the neurophysiological mechanism of nociception [17].

Although the literature on the use of amantadine in relieving neuropathic pain is scarce, studies suggest its effectiveness in modulating central sensitization [18,19]. Amantadine promoted a reduction in oxidative stress and prevented hypersensitivity in spinal cord injury in rats [20]. In cats, the combination of meloxicam and amantadine ensured clinical improvement in sciatic nerve injury [21].

In accordance with the need for a major understanding of the efficacy of amantadine in oncological neuropathy, this study aimed to clarify the antinociceptive potential of amantadine through the assessment of the mechanical nociceptive threshold, in addition to elucidating its inhibitory effect on the production of reactive oxygen species, lipid peroxidation and enzymatic activity and evaluating its anti-inflammatory effect on a vincristine-induced neuropathy model.

## 2. Materials and Methods

### 2.1. Animals and Housing Conditions

Forty-eight male Wistar rats (*Rattus norvegicus*) were used, weight ranging from 250–300 g, from the Laboratory Animal Rearing and Maintenance Station of the State University of Santa Cruz–UESC, Ilhéus, BA, Brazil. The animals were kept in the Experimental Laboratory of the UESC Veterinary Hospital in 1500 cm^2^ polypropylene boxes lined with wood shavings (containing five animals each) in a temperature-controlled environment (22 °C) and a 12 h photoperiod (light/dark), receiving commercial food and water ad libitum, with an acclimation period to the experimental conditions of one week, to equalize all environmental and food conditions, and reduce possible interference with the research results.

### 2.2. Chemotherapy-Induced Neuropathic Pain Model and Experimental Groups

Vincristine was administered at doses of 0.05 mg/kg/day intraperitoneally (IP), once a day for 14 days, to develop the experimental neuropathic pain model [22]. The negative control group (GCN) received saline solution (0.9% NaCl) intraperitoneally in a volume of 0.1 mL/animal, once a day for 14 days. Animals treated with amantadine received oral amantadine in different doses (2 mg, 5 mg, 12 mg, 25 mg or 50 mg), once a day for 14 days, one hour before the administration of vincristine sulfate.

The animals were randomly divided into eight groups (n = 6):

- Naïve group (GN): animals did not receive any drug or vehicle;
- Negative control group (GCN): saline solution only (NaCl 0.9%);
- Positive control group (GCP): vincristine sulfate only 0.05 mg/kg/day;
- Group GA2: amantadine 2 mg/kg/day + vincristine sulfate 0.05 mg/kg/day;
- Group GA5: amantadine 5 mg/kg/day + vincristine sulfate 0.05 mg/kg/day;
- Group GA12: amantadine 12 mg/kg/day + vincristine sulfate 0.05 mg/kg/day;
- Group GA25: amantadine 25 mg/kg/day + vincristine sulfate 0.05 mg/kg/day;
- Group GA50: amantadine 50 mg/kg/day + vincristine sulfate 0.05 mg/kg/day.

### 2.3. Assessment of Mechanical Nociceptive Threshold

Assessment of nociceptive mechanical threshold was implemented using a digital analgesimeter through increasing pressure method. The nociceptive mechanical threshold was defined as the minimum force necessary to trigger the paw withdrawal reflex. Withdrawal threshold was assessed through application of pressure ranging from 0 to 50 g with an accuracy of 0.2 g. Voluntary movements associated with locomotion were not considered a withdrawal response. Stimuli were applied at 5 s intervals. The measurements were repeated 3 times and the final value was obtained by average of the 3 measurements.

### 2.4. Euthanasia and Sample Collection

The animals were euthanized on the 14th day, after the mechanical nociceptive threshold evaluation, through a propofol overdose (150 mg/kg). The spinal cord was collected through hydraulic extrusion technique [23]. The lumbosacral region of the spinal cord (9 mm) was removed and segmented into three fragments (3 mm) for storage and subsequent analysis. Two fragments were stored in RNAse- and DNAse-free cryovials at −80 °C for subsequent enzymatic and PCR analysis. The third fragment was stored in 4% paraformaldehyde for 24 h with subsequent change to 70% alcohol, followed by tissue preparation, changing solutions to 80% alcohol (50 min), 90% alcohol (50 min), absolute alcohol (50 min), xylene (50 min) and paraffin (30 min, controlled temperature between 55 °C and 60 °C), with ensuing embedding in histological paraffin at the same temperature. Then, histological sections with a thickness of 4 µm were obtained and placed on previously gelatinized slides.

### 2.5. Enzyme Analyses

#### 2.5.1. Catalase Activity Analysis

The analysis of catalase activity (CAT) was carried out using a spectrophotometer device (Thermo Scientific GENESYS 6™ UV-Vis, Waltham, MA, USA) with the wavelength set at 240 nM, with readings through quartz cuvettes. For the “blank” solution, 50 mM potassium phosphate buffer was used with pH adjusted to 7, and the device was calibrated to zero. Soon after, 40 µL of a hydrogen peroxide (H_2_O_2_) solution was added, prepared using 200 µL of H_2_O_2_ and 9.8 mL of distilled water, protected from light. The reading was then collected every 15 s for 45 s; then, 9 µL of the sample was added and the reading was carried out until 150 s.

#### 2.5.2. Analysis of Superoxide Dismutase Activity

The analysis of Superoxide Dismutase (SOD) activity was also executed using a spectrophotometer (Thermo Scientific GENESYS 6™ UV-Vis, Waltham, MA, USA), using a wavelength of 420 nM, with readings through quartz cuvettes. The “blank” solution was prepared by addition of 1200 µL of TFK and 32 µL of EDTA, and the device was calibrated to zero. Soon after, 154 µL of the pyrogallol solution was added and the absorbance was read for 5 min every 30 s, to obtain the absorbance values of the pyrogallol auto-oxidation. At 5 min of the reaction, 154 µL of the sample was added to the cuvette, and readings continued every 30 s during 10 min of reaction.

### 2.6. Protein Quantification

The protein concentration of the sample was quantified using the Bradford method (1976), based on the principle of binding of the dye to the protein. The method uses bovine serum protein (BSA), calculating the protein concentration of the sample using a standard curve of BSA dilution at 0.1 mg/mL. Amounts of 150 μL of BSA at a concentration of 0.1 mg/mL and 50 μL of TFK (50 mM, pH 7.0) were placed on a plate. An amount of 10 μL of the sample was diluted in 40 μL of TFK (1:20) and then added in triplicate. An amount of 200 μL of Coomassie Blue was applied under the plate in all wells (Sigma B-0770, Sigma Aldrich Corporation, St. Louis, MO, USA). The absorbance was then read at a wavelength of 595 nm on the spectrophotometer device (SpectraMax Paradigm–Multi-Mode Detection Platform, Molecular Devices, LLC, San Jose, CA, USA).

### 2.7. Immunohistochemistry

For immunohistochemical evaluation, the streptavidin–biotin–peroxidase technique was used, using the EnVisionTM FLEX+, Mouse, High pH kit (Agilent Dako^®^, Santa Clara, CA, USA). Five histological sections from each animal were analyzed for each antibody. The protocol followed that recommended by the kit specifications. A prior standardization process appropriate to the tissue defined the time in each solution. The histological sections on the slides were heated in an oven, deparaffinized in xylene PA, rehydrated in absolute ethyl alcohol (100%, 90%, 80% and 70%, respectively), washed and immersed in citrate buffer solution (citric acid), pH 6, in a water bath at 90–98 °C for 20 min, and subsequently remained in citrate buffer for another 20 min at room temperature, followed by wash buffer washing solution (DM831, Dako^®^, Santa Clara, CA, USA).

Then, treatment started with a sequence based on Illie M. et al. (2017) [24], initially with peroxidase blocking (SM801, Dako^®^, Santa Clara, CA, USA), serum blocking (protein block) and incubation in a humidity chamber for 30 min at room temperature. The sections were soaked in the primary antibody of choice and were incubated overnight for 18 h in a humidity chamber under refrigeration at 4 °C. The utilized antibodies (Santa Cruz Biotechnology^®^, Paso Robles, CA, USA), as well as species, dilution, code and DAB development time, are described in Table 1.

The process continued the next day by washing solution, then by addition of stabilizing protein (SM804, Dako^®^, Santa Clara, CA, USA) for 30 min in a humidity chamber at room temperature, followed by washing one more time. The secondary antibody (SM802, Dako^®^, Santa Clara, CA, USA) was applied on the slides for 30 min and thereupon washed. The cromogen used was diaminobenzidine (DAB) code DM827 (Dako^®^, Santa Clara, CA, USA), in a dilution of 1:50, protected from light, with a time defined by standardization through appropriate labeling tests for each antibody. The slides were then stained in hematoxylin (HE), followed by dehydration in ethyl alcohol (70%, 80%, 90% and 100%, respectively) and xylene. Later, the slides were mounted using Canada balsam and coverslips, for later reading under an optical microscope.

### 2.8. Quantitative PCR

qRT-PCR technique was performed. Initial extraction of total RNA from the spinal cord was performed using Trizol, following the manufacturer’s instructions (Invitrogen, Life Technologies, Carlsbad, CA, USA). The method consisted of an initial stage of tissue lysis and homogenization for 5 min at room temperature to completely dissociate the nucleoprotein complexes. The lysate was transferred to a 1.5 mL microtube, and 0.2 mL of chloroform was added, followed by 15 s of homogenization, 2 to 3 min of incubation at room temperature and centrifugation at 12,000× g for 15 min at 4 °C for separation into three phases; RNA encompassed the colorless phase. In the third stage, the colorless phase was transferred to a new tube, with addition of 0.5 mL of isopropyl alcohol and incubation for 30 min at −80 °C, followed by centrifugation at 12,000× *g* for 10 min at 4 °C to precipitate the RNA. The supernatant was discarded and the pellet was placed on ice. Then, the pellets were washed with 1 mL of 75% ethanol, homogenized and centrifuged at 10,500× *g* for 5 min at 4 °C. After discarding the ethanol, the RNA pellet was solubilized in RNAse- and DNAse-free water and immediately stored at −80 °C. Analysis of the concentration and quality of RNA in the tissue of each sample was carried out using a nanodrop 2000 Spectrophotometer (Thermo Scientific, Waltham, MA, USA).

The commercial kit GoTaq^®^ qPCR 1 µg of RNA was used for reverse transcription reactions using RT-qPCR Systems (A6010, PROMEGA, Promega Corporation, Madison, WI, USA). The quantification of the target gene transcripts was conducted through qPCR using SYBR Green on the Applied Biosystems^®^ 7500 Real-Time PCR System equipment (Applied Biosystems, Foster City, CA, USA). The reactions were performed using 1.5 μL of cDNA, 100 nM of each primer and 12.5 μL of the GoTaq^®^ qPCR Master Mix reagent, 2X, in a final volume of 20 μL of reaction. The DNA amplification mix was used as a negative control and the cDNA sample replaced by water. Primers for *Grp78*, *Chop*, *Ho1*, *Perk*, *Bax*, *Bcl-xL*, *Casp 3*, *Casp 9*, *Il-6*, *Il-10*, *Il-18* and *Il-1* β were designed based on the *Rattus norvegicus* mRNA sequence (Table 2). The calculations of the gene expression were performed using the 2^−ΔΔCT^ method, and the results obtained for each group were compared quantitatively after normalization based on the expression of *Gadph Rattus norvegicus.*

### 2.9. Statistical Analyses

Statistical analysis was performed using Graph Pad Prism 5.01 software. A completely randomized design was used. Nociceptive threshold data were analyzed using two-way analysis of variance (two-way ANOVA) followed by the Bonferroni test, and the results were expressed as mean ± standard error of the mean. For immunohistochemical analysis, Student–Newman–Keuls (SNK) test was used for multiple comparisons. The ID50 of the doses used in the experiment was calculated from a dose–response curve using nonlinear regression. A significance level of 5% was adopted in all analyses.

## 3. Results

### 3.1. Effect of Chemotherapy on Mechanical Pain Hypersensitivity

**Figure 1:**
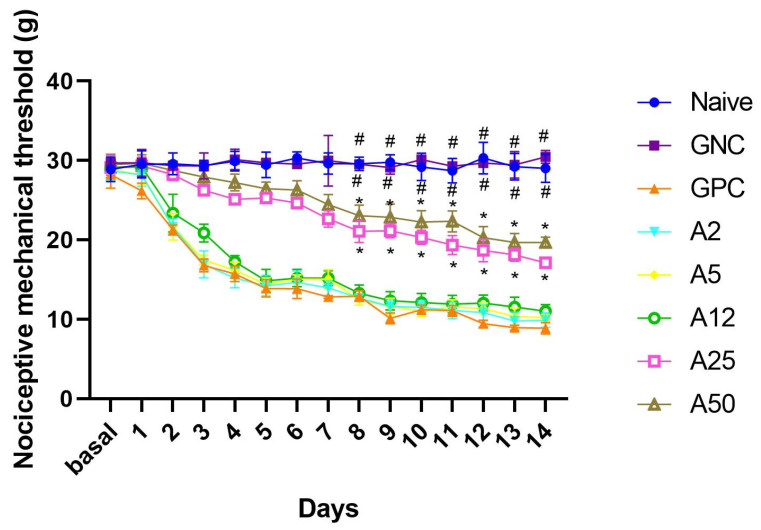
Assessment of the nociceptive mechanical threshold (g) using a digital analgesimeter, performed daily for 14 days. Values represent mean ± SEM. # (differs GPC, A2, A5, A12, A25 e A50; *p* < 0.05). * (differs GNC, naïve, A2, A5 and GPC; *p* < 0.05).

Daily administration of intraperitoneal vincristine (0.05 mg/kg) was capable of inducing mechanical hyperalgesia, evidenced by a decrease in the mechanical nociceptive threshold. Neuropathy could be observed from the fourth day (Figure 1) with a low threshold maintained in the positive control until the 14th day of the experiment, with a cumulative effect. The naïve (29.49 ± 0.19 g) and negative (29.66 ± 0.17 g) groups differed from the positive control group (14.50 ± 0.93 g), demonstrating that neuropathic pain was established through the chemotherapy drug, vincristine.

### 3.2. Amantadine Increased the Nociceptive Mechanical Threshold in Rats with Vincristine-Induced Peripheral Neuropathy

The administration of amantadine had a positive effect on preventing the development of mechanical hyperalgesia at 25 mg/kg (23.09 ± 0.62 g) and 50 mg/kg, showing a significant difference from the positive control group (24.69 ± 0.56 g). The ID50 (calculated from the effective dose capable of increasing the nociceptive mechanical threshold by 50%) was 9.58 mg/kg. The doses of 2 mg, 5 mg and 12 mg did not show a significant difference (*p* > 0.05) compared to the positive control group (14.50 ± 0.95 g) (Figure 1).

### 3.3. Amantadine Treatment Inhibits the Expression of Pro-Inflammatory Cytokines in the Spinal Cord of Rats with Vincristine-Induced Peripheral Neuropathy

**Figure 2:**
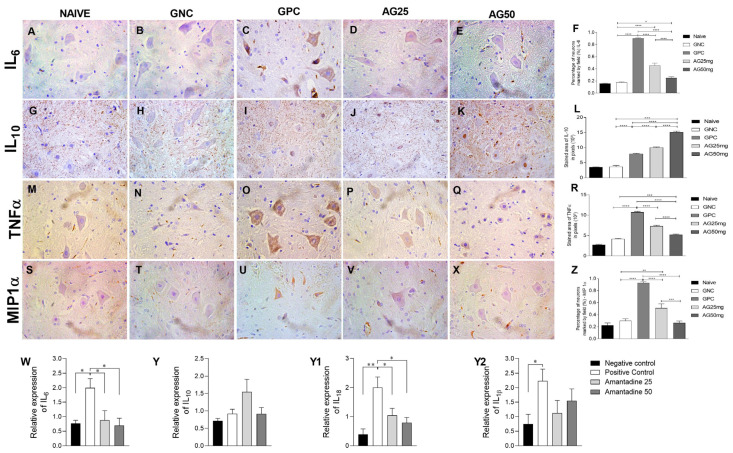
Expression of IL-6, IL-10, TNF-α and MIP-1α in the spinal cord of rats treated with amantadine for vincristine-induced peripheral neuropathy. (**A**–**E**) Photomicrographs of immunohistochemical expression of IL6 (streptavidin–biotin–peroxidase; Harris hematoxylin; 40×). (**G**–**K**) Photomicrographs of the immunohistochemical expression of IL10 (streptavidin–biotin–peroxidase; Harris hematoxylin; 40×). (**M**–**Q**) Photomicrographs of the immunohistochemical expression of TNF-α (streptavidin–biotin–peroxidase; Harris hematoxylin; 40×). (**S**–**X**) Photomicrographs of the immunohistochemical expression of MIP1a (streptavidin–biotin–peroxidase; Harris hematoxylin; 40×). (**F**) Immunolabeling area, in pixels, of IL6 expression (mean ± SEM; n = 6). (**L**) Immunolabeling area, in pixels, of IL-10 expression (mean ± SEM; n = 6). (**R**) Immunolabeling area, in pixels, of TNF-α expression (mean ± SEM; n = 6). (**Z**) Immunolabeling area, in pixels, of MIP-1α expression (mean ± SEM; n = 6). (**W**–**Y2**) Relative gene expression of IL-6, IL-10, IL-18 and IL-1β in the spinal cord (mean ± SEM; n = 6). * (*p* < 0.05), ** (*p* < 0.01), *** (*p* < 0.001), **** (*p* < 0.0001).

Pro-inflammatory cytokine expressions of IL-6, TNF-α and MIP-1α, tested through an immunohistochemical technique, demonstrated a statistically significant increase (*p* = 0.0001) in groups that received a daily intraperitoneal injection of vincristine, evidencing the increase in these cytokines in the inflammatory process of chemotherapy-induced neuropathy (Figure 2). Amantadine was able to significantly reduce (*p* < 0.0001) the expression of Il-6 in the spinal cord of rats with chemotherapy-induced neuropathy, which is demonstrated by the statistical difference in relation to the positive control group; its highest effect was observed at the dose of 50 mg/kg. Similarly, the TNF-α expression was reduced in groups treated with amantadine; the 50 mg/kg group showed lower immunostaining (Figure 2).

**Figure 3:**
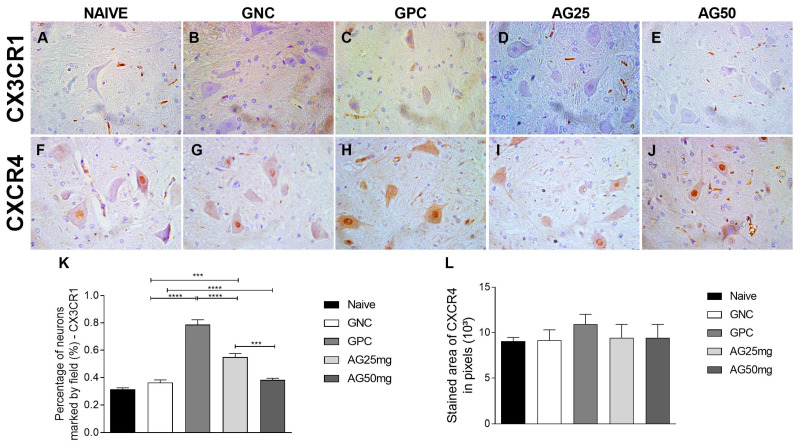
Expression of CX3CR1 and CXCR4 in the spinal cord of rats treated with amantadine for vincristine-induced peripheral neuropathy. (**A**–**E**) Photomicrographs of immunostaining expression in rat spinal cord of CX3CR1 (streptavidin–biotin–peroxidase; Harris hematoxylin; 40×). (**F**–**J**) Photomicrographs of the immunohistochemical expression of CXCR4 (streptavidin–biotin–peroxidase; Harris hematoxylin; 40×). (**K**) Immunolabeling area, in pixels, of CX3CR1 expression (mean ± SEM; n = 6). (**L**) Immunolabeling area, in pixels, of CXCR4 expression (mean ± SEM; n = 6). *** (*p* < 0.001), **** (*p* < 0.0001).

MIP-1α also showed a decrease (*p* < 0.0001) in its expression in animals treated with amantadine, with the reduction being more pronounced at the highest dose tested (50 mg/kg), suggesting a role for the macrophage inflammatory protein in the mechanism of chemotherapy neuropathy and the anti-inflammatory mechanism of amantadine. The same occurred with CX3CR1, demonstrating a dose-dependent decrease (*p* < 0.0001). In the immunostaining of the anti-inflammatory cytokine interleukin-10, an increase was identified in animals that received amantadine (Figure 3). The expression of CXCR4 immunostaining did not demonstrate a statistically significant difference between the groups (Figure 3).

SOD and CAT immunostaining expression was significantly increased in animals that received amantadine at 50 mg/kg (*p* = 0.0001), which evidences the role of amantadine in oxidative stress. The dose of 25 mg/kg did not demonstrate significant expression of SOD and CAT, suggesting an antioxidant effect only at the highest tested dose. On the other hand, GPx immunostaining did not exhibit a significant difference between the positive control group and the group that received amantadine; it was only greater in the groups that received vincristine in relation to the control groups without treatment and saline solution, suggesting an induction of antioxidant release to oppose chemotherapy-induced neuropathy.

### 3.4. Amantadine Treatment Increased the Expression of Antioxidant Enzymes (CAT and SOD) in the Spinal Cord of Rats with Vincristine-Induced Peripheral Neuropathy

**Figure 4:**
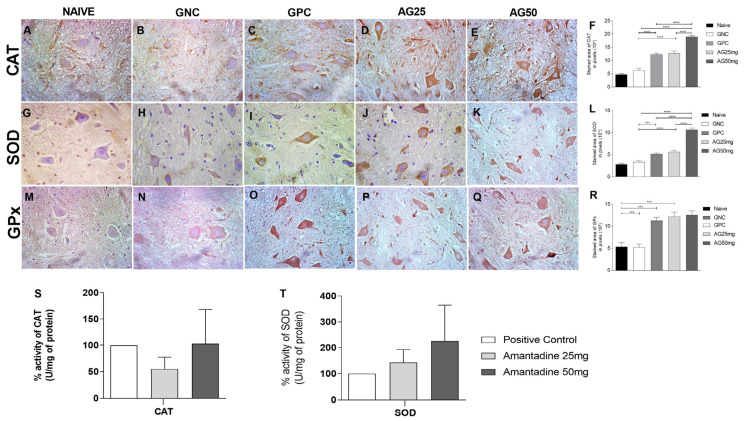
Expression of CAT, SOD and GPx in the spinal cord of rats treated with amantadine for vincristine-induced peripheral neuropathy. (**A**–**E**) Photomicrographs of immunostaining expression in rat spinal cord of catalase (CAT) (streptavidin–biotin–peroxidase; Harris hematoxylin; 40×). (**G**–**K**) Photomicrographs of immunostaining expression in rat spinal cord of Superoxide Dismutase (SOD) (streptavidin–biotin–peroxidase; Harris hematoxylin; 40×). (**M**–**Q**) Photomicrographs of immunostaining expression in rat spinal cord of Glutathione Peroxidase (GPx) (streptavidin–biotin–peroxidase; Harris hematoxylin; 40×). (**F**) Immunolabeling area, in pixels, of CAT expression (mean ± SEM; n = 6). (**L**) Immunolabeling area, in pixels, of SOD expression (mean ± SEM; n = 6). (**R**) Immunolabeling area, in pixels, of GPx expression (mean ± SEM; n = 6). (**S**) Enzymatic activity expression of CAT (mean ± SEM; n = 6). (**T**) Enzymatic activity expression of SOD (mean ± SEM; n = 6). *** (*p* < 0.001), **** (*p* < 0.0001).

Vincristine played an important role in the statistically significant decrease (*p* < 0.0001) in the antioxidant enzymes catalase (CAT) and Superoxide Dismutase (SOD) (*p* < 0.0001), which is demonstrated by the difference between the positive control group in relation to the naïve and saline solution groups (Figure 4). Treatment with amantadine proved to be effective in a dose-dependent manner, once it increased SOD and CAT (*p* = 0.0001) activity, corroborating the results obtained with immunohistochemistry (Figure 4).

### 3.5. Amantadine Treatment Inhibits the Expression of Reticular Stress Mediators in the Spinal Cord of Rats with Vincristine-Induced Peripheral Neuropathy

Vincristine-induced peripheral neuropathy in rats can cause endoplasmic reticulum stress in the spinal cord. Quantitative PCR (qPCR) was performed to verify whether treatment with amantadine at the doses used could prevent the occurrence of such. For this purpose, misfolded-protein activation-pathway key mediators, that indicate the occurrence of reticular stress, were analyzed, *Grp78*, *CHOP*, *Perk* and *Ho1*.

**Figure 5:**
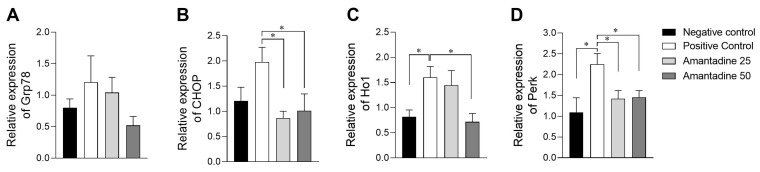
Relative gene expression graphs of *Grp78*, *CHOP*, *Ho1* and *Perk* in quantitative PCR (qPCR) technique in rat spinal cord in control groups and groups treated with amantadine at doses of 25 mg and 50 mg. (**A**) Relative gene expression of *Grp78* (mean ± SEM; n = 6); (**B**) relative gene expression of *CHOP* (mean ± SEM; n = 6); (**C**) relative gene expression of *Ho1* (mean ± SEM; n = 6); and (**D**) relative gene expression of *Perk* (mean ± SEM; n = 6). * (*p* < 0.05).

*Grp78* gene expression showed no significant difference between groups (Figure 5). As for *CHOP* expression, the 25 and 50 mg/kg treatments with amantadine induced significantly lesser values for changes caused by neuropathy, when compared to the positive control group (Figure 5, *p* < 0.01).

Peripheral neuropathy increased *Perk* gene expression (Figure 5, *p* < 0.01), compared to the negative control. Amantadine 50 mg/kg was the only dose to cause a significant reduction in its gene expression. Concerning reticular stress signal (*Ho1*) expression, treatments with amantadine 25 and 50 mg/kg significantly reduced its expression caused by neuropathy, compared to the positive control group (Figure 5; *p* < 0.001). Altogether, these data show that peripheral neuropathy up-regulates the expression of genes related to UPR and reticular stress in the spinal cord of rats; also, amantadine moderates their expression.

**Figure 6:**
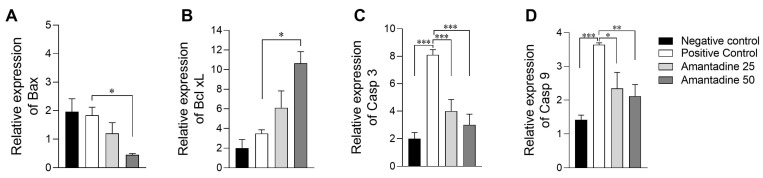
Relative gene expression graphs of *Bax*, *Bcl xL*, *Casp 3* and *Casp 9* in quantitative PCR (qPCR) technique in rat spinal cord in control groups and groups treated with amantadine at doses of 25 mg and 50 mg. (**A**) Relative gene expression of *Bax* (mean ± SEM; n = 6); (**B**) relative gene expression of Bcl xL (mean ± SEM; n = 6); (**C**) relative gene expression of *Casp 3* (mean ± SEM; n = 6); and (**D**) relative gene expression of *Casp 9* (mean ± SEM; n = 6). * (*p* < 0.05), ** (*p* < 0.01), *** (*p* < 0.001).

Since *Bcl-x* is the predominant protein of the Bcl-2 family and one of the main regulators of apoptosis, and the *Bcl-xL*/*Bax* protein ratio is a good predictor of cell survival, we also evaluated the gene expression of both markers. Peripheral neuropathy induced by vincristine (positive control group) down-regulates *Bcl-xl* expression, while up-regulating *Bax* gene expression, with a significant difference in the group treated with amantadine 50 mg/kg (Figure 6; *p* < 0.05).

Regarding the expression of *CASP 3* and *CASP 9*, groups treated with amantadine 25 mg and 50 mg/kg significantly reduced the spinal cord gene expression caused by neuropathy (Figure 6; *p* < 0.05). Accordingly, these data show that peripheral neuropathy promotes the expression of immunological and apoptotic markers and that treatment with amantadine was able to therapeutically modulate this response.

## 4. Discussion

### 4.1. Vincristine Induces Neuropathic Pain in Chemotherapy

In the present study, we demonstrated that daily intraperitoneal administration of vincristine (50 µg/kg) was able to induce mechanical hyperalgesia in rats. The dose of vincristine used in this study was based on a previous study by Nozaki-Taguchi et al., 2001 [22], who established a chemotherapy-induced neuropathy model with administration of intravenous vincristine 30–100 µg/kg for 14 days. The first measurement of the mechanical threshold occurred on the seventh day, which revealed tactile allodynia observed in doses greater than 50 µg/kg/day, in a dose-dependent manner. 

The hyperalgesia observed in the present study was also perceived in other studies conducted by Authier et al., 2003 [25], where they demonstrated sustained allodynia and mechanical hyperalgesia in rats when doses of vincristine were administered for 10 days (50, 100 and 150 μg/kg IV), by Kiguchi et al. (2008) [26], with doses of 10–100 μg/kg, by Chiba et al., 2017) [27], with doses of 100 µg/kg/day for 7 and 14 days, and by Vashistha, Sharma and Jain, 2016 [28], where they observed a reduction in the mechanical nociceptive threshold in rats with vincristine (50 ug/kg/day, IP) for 11 days.

Until the day of the present study, there were insufficient data regarding the combination of amantadine and vincristine in the same medical protocol to assess potential risks, such as side effects, the decrease in efficacy of the anti-cancer treatment, and the development of viral resistance. A study in 2020 aimed to assess the use of amantadine to evaluate the chemotherapy response in lung cancer. Systemic therapy choices were varied, including a combination of chemotherapeutic drugs such as cisplatin, carboplatin, pemetrexed and others. No toxicities or side effects were observed with the addition of amantadine to chemotherapy. However, the study did not mention the use of vincristine [29]. Nevertheless, further research is recommended to investigate potential interactions and risks associated with their concurrent use.

### 4.2. Amantadine Alleviates Mechanical Hyperalgesia in Chemotherapy-Induced Neuropathic Pain

By antagonizing NMDA receptors, modulating glutamatergic neurotransmission and inhibiting central sensitization processes that contribute to chronic pain, NMDA antagonist drugs may normalize neuronal activity and reduce the hyperexcitability associated with neuropathic pain [19]. 

Some of the primary medications utilized for pain management during cancer chemotherapy include NMDA antagonists like ketamine and methadone. Both drugs have extensive research supporting their efficacy in managing neuropathic pain, with ketamine noted for its rapid pain relief onset and effectiveness across acute and chronic conditions [30]. However, ketamine also presents risks such as psychotomimetic effects and potential for abuse, necessitating vigilant monitoring [30]. Methadone, on the other hand, offers prolonged pain relief with lower respiratory depression risks compared to other opioids, though it requires careful monitoring for drug interactions and cardiac side effects like QT interval prolongation [31].

Amantadine and memantine, while showing promise as potential treatments for neuropathic pain, lack robust evidence compared to ketamine and methadone. Both drugs are generally well tolerated, with minimal sedative effects and potential neuroprotective properties [32,33]. Memantine has demonstrated cognitive enhancement and neuroprotection in specific neurological conditions, but may cause gastrointestinal issues like nausea and diarrhea, necessitating careful dosing and monitoring [16]. Amantadine, known for its neuroprotective effects, has been used successfully in combination therapies but can lead to CNS side effects like nausea, orthostatic hypotension, dizziness and confusion, requiring adjustments in patients with renal impairment. Further research is essential to delineate their specific effects on neuropathic pain patients [34,35].

The doses used in the present study were based on previous studies in rats whose effective doses ranged from 10 mg [36] to 135 mg [37]. In our study, it was demonstrated that doses of 2, 5 and 12 mg/kg were not effective in improving mechanical hyperalgesia in chemotherapy-induced neuropathy in rats. The effective doses improving mechanical hyperalgesia were 25 and 50 mg/kg, indicating a statistical difference from the positive control group. The dosage commonly used by humans, particularly in Parkinson’s disease patients where its use is well documented, generally tolerates doses up to 200 mg of conventional amantadine. This dose range is widely considered safe and well tolerated [38]. Muller et al., in 2022 [38], demonstrated that extended-release amantadine capsules provide enhanced convenience with a steady and sustained release of amantadine over time, leading to improved tolerability and efficacy compared to traditional immediate-release formulations. The use of dosages such as 25 mg/kg/day and 50 mg/kg/day in patients with neuropathic pain remains to be elucidated regarding safety and side effects.

Previous studies with amantadine demonstrated a positive effect in different models of neuropathic pain, on both animals and humans. In a study in dogs with refractory osteoarthritis, the use of 3–5 mg/kg demonstrated effective improvements in chronic pain, associated with an NSAID (meloxicam) [39]. Previous denervation of the sciatic nerve study in rats [40] showed that 90 mg/kg promoted a reduction in nociception in a model of neuropathic pain. The drug was also effective in postoperative cognitive dysfunction in rats at a dose of 25 mg/kg/day [41]. In a study with a traumatic brain injury (TBI) model in rats, doses of 45 and 135 mg/kg were able to reduce post-TBI depression behavior [30]**.** In humans, amantadine effectively reduced pathological low-back pain in patients with neuropathic pain [32], surgical neuropathic pain in cancer patients [33] and peripheral sensitization in patients with chronic low-back pain [42]. Pud and collaborators, in 1998, concluded that, in models in which NMDA receptor activation-dependent neuropathic pain is possible, amantadine can effectively reduce the existing neuropathic pain [33].

The findings from our study also indicate that the antinociceptive effect of amantadine slightly decreased within the 14-day study period, raising questions about its long-term efficacy. Fumagalli et al., 2021, suggested that neuronal damage might precede the inflammatory process in various preclinical models of chemotherapy-induced peripheral neuropathy, potentially leading to neuropathy progression over time [43]. This progression could involve increased inflammation or neuronal injury that may not be effectively managed solely by NMDA antagonists.

### 4.3. Regulation of Inflammatory Mediators by Amantadine Demonstrates Anti-Inflammatory Effects in Chemotherapy-Induced Neuropathy 

Vincristine was able to induce an inflammatory response in the spinal cord, evidenced by an increase in immunostaining through an immunohistochemical technique in the positive control group of the pro-inflammatory cytokines IL-6, TNF-α, MIP-1α, IL-18 and IL-1 β. IL-6 has been shown to be an inflammatory cytokine with a fundamental role in pathological pain, acting on CIPN [44]. It is directly linked to nociceptive plasticity, increasing transduction in sensory neurons [45], and thus contributes to both nociceptor sensitization and central sensitization [46,47,48].

TNF-α is of great importance in pro-inflammatory processes. It is considered the main mediator in processes that involve necrosis, apoptosis or proliferation, being a suitable systemic marker of tissue damage [49,50], regulating several types of glutamatergic receptors [51,52], and is involved in the production of the neurotransmitter glutamate, as well as decreasing glutamate reuptake, raising it to toxic levels [53]. In the dorsal horn of the spinal cord, TNF-α increases glutamate release from TRPV1-expressing C-fiber terminals, leading to increased excitatory synaptic transmission on interneurons in lamina II, which in turn create synapses on lamina I projection neurons to form a pain circuit [54]. The reduction in TNF-α immunostaining in animals treated with amantadine suggests the inhibition of this circuit.

There was a decreased expression of MIP-1α, in animals treated with 25 and 50 mg/kg of amantadine in dorsal medulla neurons. Macrophage inflammatory protein is an inflammatory chemokine that sensitizes TRPV1, which is sensitive to noxious stimuli receptors and is an important mediator in pathological pain, through its receptor CCR1, suggesting that this chemokine may participate in neuropathic pain through glial cell crosstalk [55]. Likewise, in a model of neuropathy induced by sciatic nerve ligation, an increase in MIP-1α was observed in animals with CIPN [56].

Amantadine’s ability to regulate inflammatory mediators contributes to its neuroprotective effects. By mitigating the inflammatory response, it prevents further neuronal damage and promotes the survival and function of neurons, which is critical in managing chronic pain conditions like CIPN [14,15,16,17,18,19]. In the present study, the administration of amantadine 25 and 50 mg/kg proved to effectively reduce pro-inflammatory cytokine expression (IL-6, TNF-α, MIP-1α, IL-18 and IL-1β), and increase cytokine anti-inflammatory IL-10 expression in the spinal cord of rats treated daily. This proposes an anti-inflammatory effect of amantadine in CIPN. Similarly, in a rat spinal cord injury model, amantadine exhibited a protective effect with reduced neuroinflammation and oxidative stress [57] in post-operative rats [33], and in a sepsis model induced by cecal ligation and puncture [58]. Milligan et al., 2006 [59] confirmed that the pharmacological application of Il-10 had positive results attenuating hyperalgesia and allodynia behaviors across various pain models, demonstrating its effectiveness in alleviating pain symptoms.

Our results demonstrate that CX3CR1 expression was up-regulated in the spinal cord of vincristine-treated animals, whereas it was down-regulated by amantadine treatment, in a dose-dependent manner. The same occurred with a study using mitokine in the chemotherapy neuropathy model with vincristine [60]. CX3CR1 is expressed in the dorsal horn of the spinal cord and mediates neuron–glia communication; in the case of peripheral neuropathy with interruption of homeostasis, it increases signaling in the dorsal horn of the spinal cord, thus increasing maladaptive neuron–glia signaling. This contributes importantly to the amplification of nociceptive transmission during the neuropathy process [61].

The alpha-chemokine receptor (CXCR4) participates in the development and maintenance of neuropathic pain mediating the transport of progenitor cells in the bone marrow [62], having also demonstrated the modulation of neuropathic pain through the activation of the ERK pathway [63]. Our results demonstrate that there was no statistically significant difference between the groups. In the same way, Deng et al., 2012 [64], in a chemotherapy-induced neuropathy model with cisplatin and paclitaxel, concluded that CXCR4 signaling did not contribute to the maintenance of chemotherapy-induced neuropathy, and that blocking CXCR4 signaling with AMD3100 failed to reverse the established chemotherapy-induced neuropathy [65].

The neuroinflammatory modulation induced by amantadine at doses of 25 and 50 mg/kg in this study points to an important therapeutic target in the management of CIPN, in view of the fact that, with these glial mediators (IL-6, TNF-α, MIP-1α and IL-10), CX3CR1 can pre-eminently modulate excitatory and inhibitory synaptic transmission, leading to sensitization and enhanced chronic pain states.

### 4.4. The Treatment with Amantadine Was Effective in Increasing Antioxidant Enzymes in the Cord of Rats with Vincristine-Induced Peripheral Neuropathy

Vincristine significantly decreased antioxidant enzymes catalase (CAT) and Superoxide Dismutase (SOD). This is in agreement with previous studies that identified a relationship between CIPN induced by vincristine and oxidative stress [66,67,68,69]. 

Damage to peripheral nerves may involve oxidative stress mediated by mitochondria, damage to the myelin sheath and other antioxidant enzymes, and the explanation of the mechanisms by which the oxidative stress pathway acts in neuropathic pain may be useful to increase the chances of therapeutic protocol improvement for CIPN patients [70]. SOD participates in the process of neuropathic pain, since the superoxide anion radical, produced via xanthine oxidase, and nitric oxide, as a precursor of peroxynitrite in NMDA, are involved in central sensitization mediation [71].

Treatment with amantadine was effective in increasing SOD and CAT. In accordance with our findings, Mata-Bermudez et al., in 2021 [20], demonstrated the role of amantadine in oxidative stress in a model of spinal cord injury in rats. Orhan et al., in 2021 [72], obtained a similar result under oxidative stress in a model of ischemia and reperfusion injury in the hind limb of rats, using amantadine. In a model of traumatic spinal cord injury in rats, Dogan and Karaka in 2020 [57] had similar effects with the administration of amantadine, inhibiting oxidative stress and leading to improvement in neuropathic pain.

Dogan and Karaka, 2020 [57] and Orhan et al. 2021 [72] observed an increase in *GPx* expression in models of spinal cord injury and ischemia and reperfusion injury in the hind limb of rats, respectively. In the present study, this finding could not be identified, as the treated groups did not obtain a significant statistical difference between them, which demonstrates that the amantadine antioxidant pathway releases antioxidant enzymes in chemotherapy-induced neuropathy, with a significant increase in SOD and CAT in the tested groups, but not GPx, thus encouraging further research based on this pathway for onward elucidation. Although there was no significant difference between the groups, there was a trend towards increased antioxidant activities. Other factors may have influenced the quantification of sample activity, such as the presence of endogenous inhibitors or activators (Figure 4).

### 4.5. Amantadine Treatment Inhibits the Expression of Reticular Stress Mediators in the Spinal Cord of Rats with Vincristine-Induced Peripheral Neuropathy 

In this peripheral neuropathy model, vincristine was found to contribute to inducing endoplasmic reticulum stress, as evidenced by the increased gene expression of *CHOP*, *PerK* and *Ho1* in the quantitative analysis of PCR, as observed in other studies of neuropathic pain-induced reticulum endoplasmic stress in the rat spinal cord [73]. Treatment with amantadine at doses of 25 mg/kg and 50 mg/kg was able to reduce the gene expression of *CHOP* and *Ho1*, and only the dose of 50 mg/kg was able to reduce *PerK*. Similarly, a study that sought to demonstrate the effectiveness of ketamine, another NMDA antagonist, on endoplasmic reticulum stress in the process of neuropathic pain concluded that the drug was effective in reducing ER stress markers, suggesting that there may be a relationship with the NMDA pathway [74].

### 4.6. Modulatory Effect of the Spinal Cord Response to the Apoptotic Mechanisms of Amantadine in Chemotherapy-Induced Neuropathy

The process of apoptosis is one of the main pathways for neuron death during spinal cord injury [75]. In this process, caspases, and proteins from the *Bcl-2* family, such as *Bcl-x* (pro-survival protein), play a major role in determining cell death or survival. Considering that *Bcl-x* is the predominant protein of the Bcl-2 family, one of the main regulators of apoptosis, and that the *Bcl-xL/Bax* protein ratio is a good predictor of cell survival, we also evaluated the gene expression of these markers. Vincristine-induced peripheral neuropathy (positive control group) reduced *Bcl-xl* expression and increased *Bax* gene expression, which corroborates studies of apoptosis in spinal cord injury [76]. The expressions of *CASP 3* and *CASP 9* were also higher in the untreated neuropathy group, while there was a significant decrease in the expression of these caspases in spinal cord tissue affected by vincristine after treatment with amantadine. Similarly, in a study involving glutamate [77], the use of amantadine led to a decrease in the apoptotic index. This indicates its potential in minimizing cell death in the cerebral cortex.

## 5. Conclusions

Amantadine demonstrated efficacy in reducing mechanical hyperalgesia in animals treated with 25 and 50 mg/kg/day, in a dose-dependent manner. It presented an anti-inflammatory effect through the activation of anti-inflammatory cytokines and decreased expression of pro-inflammatory cytokines. An antioxidant effect was demonstrated through the increased expression of the antioxidant enzymes SOD and CAT, and regulation of apoptotic mediators. Our data indicate that amantadine may be a potential alternative for the treatment of vincristine-induced peripheral neuropathic pain, although the study suggests that further investigation is needed regarding drug interactions, dosing, effectiveness in humans and potential side effects.

## Figures and Tables

**Table 1:** Antibodies used in immunohistochemistry and respective dilutions, development time and code.

Antibody	Dilution	DAB Time	Code
Anti-CX3CR1	1:250	10 min	anti-CX3CR1, sc-377227
Anti-CXCR-4/IgC	1:200	5 min	anti-CXCR-4, sc-53534
Anti-Catalase	1:200	7 min	anti-CAT, sc-271803
Anti-GPx	1:200	10 min	anti-GPx, sc-133152
Anti-IL-10	1:2500	3 min	anti-IL10, sc-365858
Anti-IL-6	1:1500	3 min	anti-IL6, sc-28343
Anti-MIP-1α	1:100	12 min	anti-MIP-1α, sc-36569
Anti-SOD	1:8000	15 s	anti-SOD, sc-101523
Anti-TNF-α	1:500	3 min	anti-TNFα, sc-33639

**Table 2:** List of genes and nucleotide sequences of primers for qRT-PCR.

Genes	Starters	No^Access^
*Grp78*	Forward: TGAAGGGGAGCGTCTGATTG Reverse: TCATTCCAAGTGCGTCCGAT	NM_013083.2
*Chop*	Forward: TGGCACAGCTTGCTGAAGAG Reverse: TCAGGCGCTCGATTTCCT	NM_001109986.1
*Perk*	Forward: GGCTGGTGAGGGATGGTAAA Reverse: TTGGCTGTGTAACTTGTGTCATC	NM_031599.2
*Ho1*	Forward: CAGCATACGTAAAGCGTCTCCA Reverse: CATGGCCTTCTGCGCAATCTTCTT	NM_012580.2
*Bax*	Forward: GCACGTCTGCGGGAG Reverse: ATCTGTTCAGAGCTGGTGGG	NM_017059.2
*Bcl-xl*	Forward: AGAACCTGGACTCAGACCTTC Reverse: TCCAGGATCCAAAGCCAAGA	XM_039104291.1
*CASP 3*	Forward: GAGCTTGGAACGCGAAGAAA Reverse: AGTCCATCGACTTGCTTCCA	NM_012922.2
*CASP 9*	Forward: TCCCCACTGATCAAGTCTCCT Reverse: CCAGGCTCACTTAGCAAGGAA	NM_031632.2
*IL-6*	Forward: GACTTCCAGCCAGTTGCCTTR Reverse: AAGTCTCCTCTCCGGACTTGT	NM_053595.2
*IL-1* *β*	Forward: GCACAGTTCCCCCAACTGGTA Reverse: TGTCCCGACCATTGCTGTTT	NM_ 031512.2
*IL-10*	Forward: ACCACTTTGGCAGACTTCCT Reverse: ACACAGGCGGGTTTCTTTTG	NM_053595.2
*IL-18*	Forward: GACTTCCAGCCAGTTGCCTTR Reverse: AAGTCTCCTCTCCGGACTTGT	NM_019174.4
*Gapdh*	Forward: GCGCTACAGCGGATTTTTGA Reverse: GAAGGCATACACGGTGGACT	NM_031797.2

## Data Availability

The datasets used and/or analyzed during the current study are available from the corresponding author on reasonable request.
